# Choroidal thickness in older patients with central serous chorioretinopathy

**DOI:** 10.1186/s40942-016-0046-7

**Published:** 2016-09-15

**Authors:** Caio V. Regatieri, Eduardo A. Novais, Lauren Branchini, Mehreen Adhi, Emily D. Cole, Ricardo Louzada, Mark Lane, Elias Reichel, Jay S. Duker

**Affiliations:** 1 New England Eye Center, Tufts Medical Center, 800 Washington Street, Boston, MA 02111 USA; 2 Department of Ophthalmology, Federal University of São Paulo, São Paulo, Brazil; 3 Department of Ophthalmology, Federal University of Goiás, Goiânia, Brazil; 4 Queen Elizabeth Hospital Birmingham, University Hospitals Birmingham NHS Foundation Trust, Birmingham, UK

**Keywords:** Central serous chorioretinopathy, Optical coherence tomography, Choroid

## Abstract

**Background:**

To investigate the choroidal thickness in older patients with central serous chorioretinopathy (CSCR) compared to age-matched normal subjects.

**Methods:**

Fifteen patients (30 eyes) with CSCR, all aged ≥60 years, and 21 age-matched normal subjects (21 eyes) underwent high-definition raster scanning using SD-OCT. Both eyes from CSCR patients were included in the analysis. The eyes in patients with CSCR were divided into two groups: active CSCR (17 eyes) if there was foveal-involving subretinal fluid and inactive contralateral eye group (13 eyes). Choroidal thickness was measured from the posterior edge of the retinal pigment epithelium to the choroidal–scleral junction at 500 µm intervals up to 2500 µm temporal and nasal to the fovea (11 locations).

**Results:**

The mean age of the patients with CSCR was 68.87 ± 6.83 years (mean ± standard deviation). Reliable measurements of choroidal thickness were obtainable in 70.6 % of eyes examined. The choroid was statistically significantly thicker in eyes with both active CSCR (*P* < 0.001) and inactive contralateral eyes (*P* < 0.01) when compared to normal age-matched eyes. The subfoveal choroid was 95 µm (*P* < 0.01) thicker in eyes with active CSCR (338.05 ± 31.42 µm) compared with normal eyes (243.05 ± 13.39 µm). The subfoveal choroid thickness in the inactive contralateral eyes was numerically greater than normal, and it was not statistically significantly thicker compared to the normal eyes (difference—55.68 µm, *P* > 0.05).

**Conclusion:**

Choroid in older patients with active CSCR was thicker than the choroid in age-matched normal eyes. It is important to consider CSCR as a differential diagnosis of serous retinal detachment in elderly patients with thickened choroid and to consider SD-OCT as an imaging modality by which to evaluate the choroidal thickness.

## Background

Central serous chorioretinopathy (CSCR) is a disease characterized in its active phase by a serous detachment of the neurosensory retina. On optical coherence tomography (OCT), active CSCR appears as an elevation of the full thickness neurosensory retina from the highly reflective retinal pigmented epithelium (RPE) separated by an optically empty zone. This condition has been linked to increased choroidal vascular permeability [1]. Studies using indocyanine green (ICG) angiography support this hypothesis and typically show broad and or multi-focal areas of choroidal hyperpermeability surrounding active RPE leaks [2–4]. This vascular hyper-permeability not only increases the choroidal vascular pressure but is believed to be a causative factor that leads to damage in the RPE, causing pigment epithelial detachment and finally, serous retinal detachment [5].

Recent investigations using two different spectral domain optical coherence tomography (SD-OCT) devices, Cirrus (Carl Zeiss Meditec, Inc., Dublin, CA) [6] and Spectralis (Heidelberg Engineering, Heidelberg, Germany) [7], demonstrated an increased choroidal thickness in patients with acute CSCR. Using Spectralis (Heidelberg Engineering) the mean subfoveal choroidal thickness in 28 eyes of 19 patients with CSCR (505 ± 124 µm; interquartile range, 439–573 µm) was 214 µm (85 %) greater than the previously reported mean subfoveal choroidal thickness of normal eyes (*P* ≤ 0.001).

Although CSCR typically occurs in young healthy male individuals, this condition can occur in older patients as well [8–10]. In such cases, a diagnostic and therapeutic challenge may exist since CSCR, choroidal neovascularization secondary to neovascular age-related macular degeneration (nAMD) and polypoidal choroidal vasculopathy (PCV) can all cause serous retinal detachment [11–14]. More recently, in a new hypothesis that proposes to unify CSCR and PCV entities into a single disease spectrum, CSCR has been described along with PCV as a late-stage manifestation of pachychoroid disease of the macula [15].

This study was designed to measure the choroidal thickness in patients older than 60 years with CSCR compared to age-matched normal subjects.

## Methods

### Subjects

A retrospective analysis was performed on 30 eyes of 15 patients with CSCR older than 60 years of age, and 21 eyes of 21 age-matched normal patients, who underwent high-definition (HD) 1-line raster scanning using Cirrus HD-OCT at the New England Eye Center, Tufts Medical Center, Boston, Massachusetts, between January 2010 and June 2011. The age-matched normal subjects had normal visual acuity and did not have any retinal or choroidal pathology on ophthalmoscopy.

The diagnosis of CSCR was based on history, clinical examination, fluorescein angiography, ICG angiography and SD-OCT findings. Eyes that had subretinal fluid were defined as having “active” CSCR. Eyes with active CSCR that manifested fluid for more than 4 months or presented more than two recurrent episodes were further defined as having active, chronic CSCR.

All patients had both eyes scanned with SD-OCT. Two patients (2 of 15 patients (11.8 %)] presented with active CSCR in both eyes during the study period and both eyes were included in the analysis as active, acute CSCR. The other patients [13 of 15, 26 of 30 eyes (88.2 %)] presented with active CSCR in only one eye. In these patients, the contralateral eyes were included as the “inactive contralateral eye” group. Patients with other ocular disease and uncontrolled systemic disease, phakic patients with a spherical equivalent ≥ ±3.00 diopters, and pseudophakic patients with myopic fundus were excluded from the analysis were excluded from this study.

### Choroidal thickness measurement

The scan pattern used on Cirrus HD-OCT was the HD 1-line raster. It is a 6-mm line consisting of 4096 A-scans. Images were taken with the vitreoretinal interface adjacent to the zero-delay. The HD 1-line raster has 20 B-scans averaged together without tracking. To be included in this study, images had to be at least 6 out of 10 in intensity and taken as close to the fovea as possible, which would lead to imaging at the thinnest point of the macula. However, it is important to note that slight differences in positioning could affect the measured thicknesses. Enhanced depth imaging (EDI) was not employed as it was not available on the Cirrus SD-OCT during the study period.

Using the Cirrus linear measurement tool, 2 independent observers measured choroidal thickness perpendicularly from the outer edge of the hyper-reflective RPE to the sclerochoroidal junction at 500-µm intervals temporal and nasal from the fovea, up to 2500 µm (11 locations). Furthermore, the central foveal thickness was also measured at this time in order to determine the correlation between central retinal thickness and subfoveal choroidal thickness in the eyes with active CSCR.

### Statistical analysis

Data are expressed as mean ± standard error of the mean (SEM). Statistical analyses were performed using one-way analysis of variance (ANOVA) followed by post test comparison with Bonferroni’s Multiple Test. Pearson correlation coefficient was used to evaluate the correlation between the subfoveal choroidal thicknesses and central foveal thickness. A 95 % confidence interval and a 5 % level of significance were adopted; therefore, the results with a *P* value less than or equal to 0.05 were considered significant. All statistics were calculated using Graph Pad Prism 5.0 software for Macintosh.

## Results

Seventeen eyes of 15 patients (11 men and 4 women) with active CSCR and 13 inactive contralateral eyes (without CSCR) were retrospectively studied. Patient age ranged from 60 to 79 years (mean ± standard deviation, 68.87 ± 6.83 years). Six patients (6 eyes) presented with acute active CSCR and 9 patients (11 eyes) were diagnosed as chronic active CSCR. The most common systemic condition was systemic hypertension [7 patients (46 %)]. One patient had a history of treated breast cancer and another had previously treated prostate cancer; both had no known ocular metastases. One of the patients was concurrently using inhaled corticosteroids for asthma. Additionally, 21 age-matched normal subjects (mean ± standard deviation, 64.59 ± 8.79 years) without any ocular or systemic disease were evaluated.

**Fig. 1:**
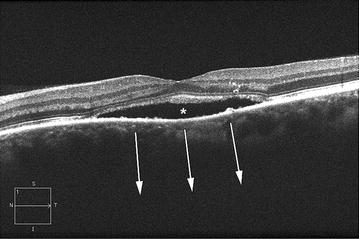
High definition 1-line optical coherence tomography B-scan image from a 72-year-old man with active central serous chorioretinopathy. Serous detachment of the neurosensory retina (*asterisk*). In this eye the choroid may be so thick that it is not possible to visualize the choroidal-sclera interface (*arrows*)

Reliable measurements of choroidal thickness were obtainable in 70.6 % of the eyes with active CSCR (12 of 17 eyes) and in 84.6 % of the contralateral eyes (11 of 13 eyes). Measurements were considered reliable if the choroidal–scleral junction could be measured. In the eyes where the choroidal–scleral junction could not clearly be identified, the extent of choroidal thickness may cause a loss of signal penetration and intensity at increasing depths because of sensitivity roll-off distal to the zero-delay line (Fig. 1). Furthermore, signal degradation could be secondary to fibrin deposition within the serous detachment or due to cataracts. All such eyes had choroidal thickness in excess of 450 µm.

**Fig. 2:**
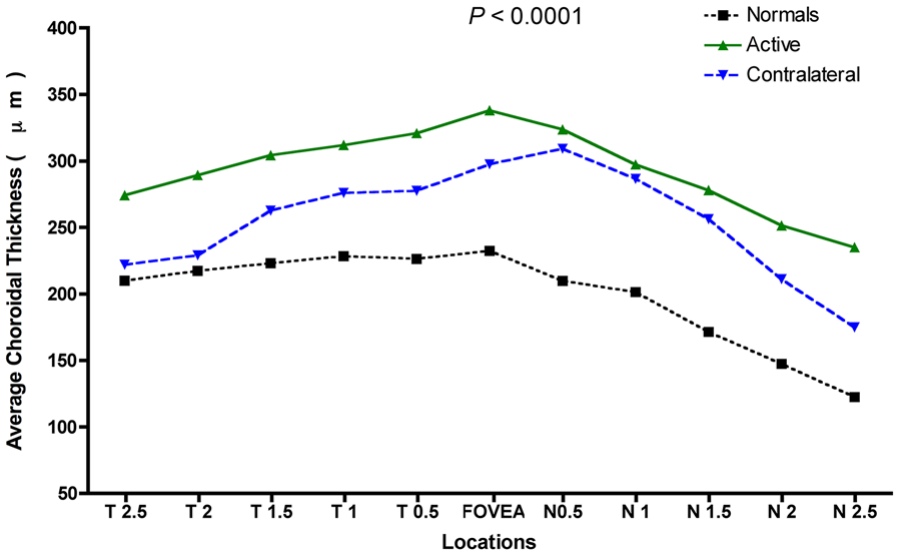
Graph of mean choroidal thickness in normal eyes, eyes with active central serous chorioretinopathy (CSCR) and inactive contralateral eye. Mean choroidal thickness at each of the 11 locations measured at 500 µm (0.5 mm) intervals temporal (T) and nasal (N), centered on the fovea. *Active*: eyes with active CSCR; *contralateral*: inactive contralateral eyes. P value represents the result of statistical analyses (ANOVA)

**Fig. 3:**
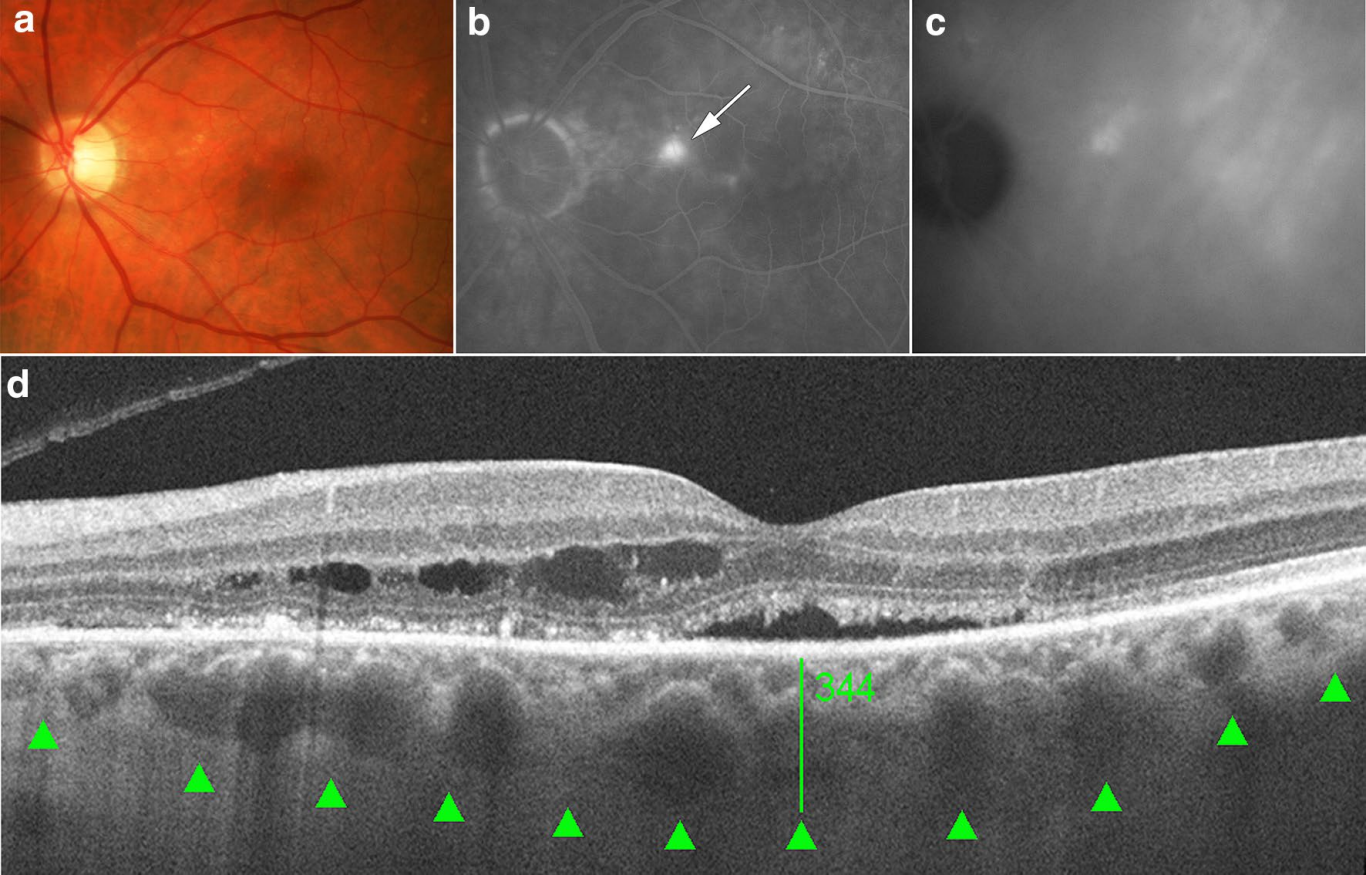
Representative images of an elderly patient with active chronic central serous chorioretinopathy (CSCR) for 8 months. **a** Fundus color photograph shows pigmentary changes on the macula area, which corresponds to a leakage point on the dye-based angiographies. **b** Fluorescein angiography image shows a focal area of leakage (*white arrow*). **c** Late phase of an indocyanine green angiography shows multiple areas of choroidal hyperpermeability. **d** SD OCT foveal B-scan shows subretinal and intraretinal fluid. The *perpendicular green line* was drawn from posterior edge of retinal pigment epithelium to choroidal–scleral junction to demonstrate the measurement. The *green arrow heads* point the choroidal–scleral junction

Mean choroidal thickness at each location was plotted in Fig. 2. The choroidal thickness in the eyes with active CSCR (P < 0.001) and the contralateral eyes (P < 0.01) was significantly thicker when comparing with normal age-matched eyes. Moreover, eyes with the active CSCR presented with a thicker choroid when compared to the contralateral eye. (P < 0.05). Representative images are shown in Fig. 3.

Table 1 shows the average choroidal thickness in each location. The choroid was noted to be thinnest nasally, thicker in the subfoveal region, and then thinner again temporally (however, not as thin as the choroid proximal to the disc). This pattern was observed in the eyes with active CSCR and in the inactive contralateral eyes. Similar results were also observed in normal subjects [16–19].

**Table 1:** Mean choroidal thickness at each of the 11 locations

Location (mm from fovea)	Mean choroidal thickness (µm)			*P*
	Normals	Active	Contralateral	
Temporal (2.5)	193.72 ± 14.45	274.22 ± 26.58	221.90 ± 24.47	0.047
Temporal (2.0)	217.05 ± 9.42	289.30 ± 27.70	229.00 ± 29.47	0.031
Temporal (1.5)	225.34 ± 9.60	304.35 ± 24.05	262.60 ± 32.51	0.013
Temporal (1.0)	231.58 ± 10.46	312.00 ± 28.39	276.00 ± 35.91	0.017
Temporal (0.5)	233.96 ± 14.25	320.80 ± 27.49	277.44 ± 38.67	0.013
Subfoveal	243.05 ± 13.39	338.05 ± 31.42	297.40 ± 33.74	0.001
Nasal (0.5)	236.29 ± 13.60	323.70 ± 23.15	309.09 ± 39.70	0.0009
Nasal (1.0)	226.34 ± 12.16	297.30 ± 21.86	286.45 ± 35.48	0.002
Nasal (1.5)	197.90 ± 12.84	278.00 ± 22.56	256.27 ± 31.44	0.0004
Nasal (2.0)	169.00 ± 14.56	251.40 ± 25.98	211.00 ± 23.13	0.0003
Nasal (2.5)	143.67 ± 13.03	235.00 ± 31.49	174.90 ± 19.20	0.0002

*Active*: eyes with active central serous chorioretinopathy; *contralateral*: inactive contralateral eyes. P values represent the results of statistical analyses (ANOVA)

**Fig. 4:**
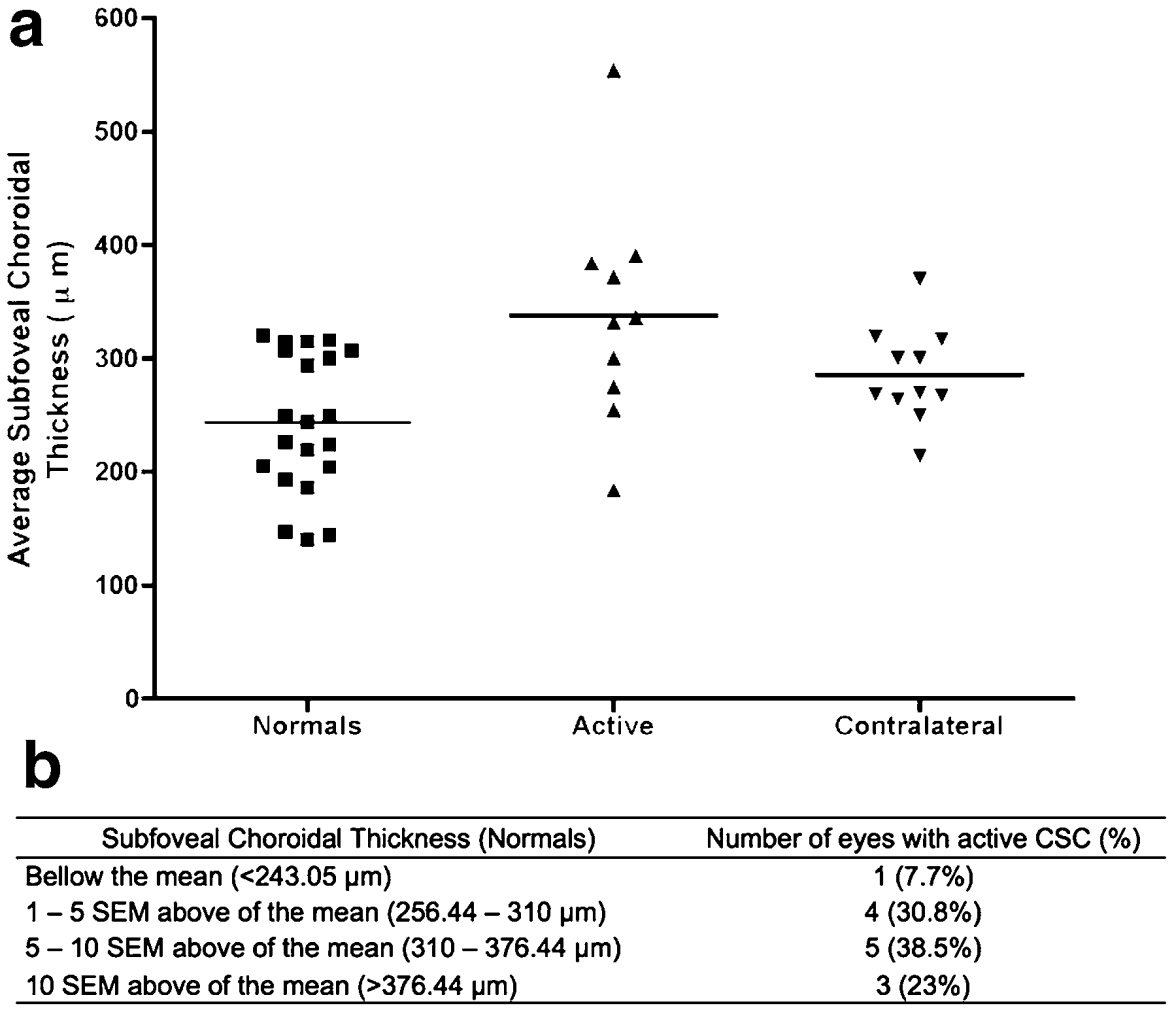
**a** Scatter plot of subfoveal choroidal thickness in normal eyes, eyes with active central serous chorioretinopathy (CSCR) and the inactive contralateral eye. *Active*: eyes with active CSCR; *contralateral*: inactive contralateral eyes. **b** Distribution of subfoveal choroidal thickness of eyes presenting with active CSCR, comparing with normal eyes. *SEM* standard error of the mean

The subfoveal choroidal thickness was noted to be increased in the eyes with active CSCR (338.05 ± 31.42 µm) compared with normal eyes (243.05 ± 13.39 µm), difference of 95.00 µm, *P* < 0.01 (Fig. 4). The subfoveal choroid thickness in the inactive contralateral eyes was numerically greater than normal, and it was not statistically significantly thicker compared to the normal eyes (difference—55.68 µm, *P* > 0.05).

The correlation between central foveal thickness of the retina and subfoveal choroidal thickness was also examined. No statistical correlation between these 2 measurements was noted in the eyes with active CSCR (r = 0.2115, *P* = 0.487) suggesting that retinal thickness may not be directly related to choroidal thickness in this group.

## Discussion

CSCR occurs most commonly in men aged 30–50 years of age [20]. However with the aging of the population and advances in posterior segment imaging, the number of older patients with the diagnosis of CSCR appears to be either increasing or our ability to detect is now more sensitive [8]. In patients over the age of 60, neurosensory detachments may occur as a result of several chorioretinal diseases, most notably nAMD, which makes diagnosing this condition in this age group a diagnostic challenge [8, 11].

Several studies suggest that an increase in the permeability of the choriocapillaris might be the primary cause of damage to the overlying retinal pigment epithelium, which would not be able to pump in a retinochoroidal direction [3, 21]. This theory supports previous studies, which have shown increased choroidal thickness in young patients. However, these studies evaluated patients with an average age less than 60 years.

This study investigates the choroidal thickness of patients older than 60 years (mean ± standard deviation, 68.87 ± 6.83 years) with active CSCR. In these patients the choroid was found to be thicker in the eyes with subretinal fluid (active CSCR) and in the inactive contralateral eye, when compared with age-matched normal eyes. In a recent publication with a younger group of CSCR patients, Chung et al. showed that the subfoveal choroidal thickness and the thickness of Haller layer were increased in the affected and the unaffected fellow eyes compared to healthy subjects [22]. This finding is important for two reasons. First, noting a thickened choroid in a patient with neurosensory retinal detachment could help differentiate CSCR and PCV from nAMD in older adults [23, 24]. It is well known that choroidal thinning occurs in normal eyes as we get older. It is believed that over the age of 50 the choroidal thickness ranges from 203.6 to 287 µm, it is also believed that this value continues to decrease by 14–15.6 µm every 10 years [17, 18, 25]. The choroid is also significantly thinner in patients with nAMD [23, 24, 26, 27]. Based on these studies, older patients with subretinal fluid, but no other manifestations of nAMD who have a choroidal thickness greater than 300 µm appear more likely to have CSCR. However, it is not possible to differentiate CSCR from nAMD based on the choroidal thickness alone. Information which can be acquired from a clinical examination and dye-based angiography are critical to make this distinction. Second, the OCT findings provide additional evidence that CSCR in adults older than 60 years may be caused by increased hydrostatic pressure in the choroid. Based on our results, we propose a cut-off value of 320 µm, above which the choroid can be considered thickened in patients older than 60 years. In this study, we did not note any normal subjects with a choroidal thickness above this value.

As aforementioned, CSCR has been described along with PCV as a late-stage manifestation of pachychoroid disease of the macula [15]. Dansingani et al. [28] analyzed the swept-source OCT *en face* imaging of patients with different diseases of the pachychoroid spectrum and noted that dilated choroidal vessels were also seen in eyes without manifest pathology, suggesting that pachychoroid is a bilateral phenomenon and that it may have a systemic basis. In a recent report, Lehmann et al. [29] have proposed that the pachychoroid morphology may have a genetic basis with a dominant mode of inheritance. This theory is in line with our results, which showed that both eyes with active CSCR and eyes with inactive CSCR presented a significantly thickened choroid compared to age-matched normals.

The measurement of the choroid was not possible in 5 eyes (29.4 %) with active CSCR and in two inactive contralateral eyes (15.4 %). This is likely due to a loss of signal penetration and intensity due to the increased thickness of the choroid. The SD-OCT imaging system is most sensitive to signals coming from reflectors close to the zero-delay; as a reflector is moved away from the zero-delay, the system will become less sensitive to the back-reflected signals [7]. Improvements in EDI SD-OCT allow for better visualization of choroidal features. This methodology works by displacing the instrument more forward than in normal OCT imaging. The inverted image allows the choroid to be located closer to the zero-delay, improving visualization [16]. However, this technology was not present on the Cirrus SD-OCT during the study period. In this study a higher percentage of patients (70.6 %) with active CSCR could be measured when compared with previous study from our group that evaluated younger patients with CSCR (30.8 %) [6]. This difference may be explained by the signal degradation secondary to the densely retinal pigmented epithelium of younger patients which was noted in previous investigations.

Previous studies showed that choroidal thickness presents a significant circadian pattern with higher values at night and lower values during the day in young adults [30–32]. However, It’s worth noting that diurnal fluctuation of choroidal thickness could not be accessed due to the retrospective nature of our study.

## Conclusion

In conclusion, this study showed that the choroid in older patients with active CSCR was thicker than the choroid in age-matched normal eyes. It is important to consider CSCR as a differential diagnosis in elderly patients and to consider SD-OCT as an imaging modality by which to evaluate the choroidal thickness. Choroidal thickness should be considered when making a diagnosis of CSCR, especially in the older population in which determining the underlying cause of serous retinal detachment is challenging.

## Abbreviations

CSCR: central serous chorioretinopathy
OCT: optical coherence tomography
RPE: retinal pigmented epithelium
ICG: indocyanine green
SD: spectral domain
PCV: polypoidal choroidal vasculopathy
HD: high-definition
IRB: Institutional Review Board
EDI: enhanced depth imaging
SEM: standard error of the mean
ANOVA: analysis of variance
mm: millimeters
µm: microns
nAMD: neovascular age-related macular degeneration
